# Modern Sociology

**Published:** 1900-05-12

**Authors:** 


### Modern Sociology.

### WOMEN AND BUSINESS LIFE.

A writer in the *Ladies' Home Journal*, which is a much more serious periodical than English magazines of the same class, declares that a reaction has set in from the wave that carried so many women out of the home circle into business life. They have most of their woman movements earlier in America than we have them here, so we may anticipate that something of the same kind is in store for us. The writer declares that the way that women have been competing with men in so many departments of industry has been fatal to their nerves and general health, has never been congenial to the majority, and has tended to bring down wages all along the line.

In all this there is much truth. For certain employments a woman is specially fitted by nature. These are domestic work, the teaching of young children, and the nursing of the sick; in all of which the maternal instinct is predominant. Add to these dressmaking and similar arts, and the lighter branches of house-decoration, where vanity and taste come in, and we have the full number of those occupations to which women would fly even if neither fashion nor necessity compelled them. It is doubtful if ever woman born hankered after being a clerk; yet there are hundreds of women doing clerks' work because they have an education which, as they think, sets them above domestic service. Certain conventions have established themselves which lay it down as a hard and fast rule that certain tasks are within the province of a lady, and others are not; and you may be sure that when such a rule has come into vogue the women whose claim to ladyhood is most doubtful will be the most determined to secure the tasks for which superior gentility is claimed.

Of course more than one influence has tended to send English and American women out into the business world in greater numbers than their sisters elsewhere. For one thing, there is the improvident habit of the Anglo-Saxon father, who lives up to his income, lives luxuriously perhaps, and has nothing to leave his daughters at his death. He trusts to their marrying, though, like the born sentimentalist he is, he has done nothing to put them in the way of getting husbands. No dowries, no husband-hunting for him. He settles his sons in business, but leaves his daughters to chance. Formerly the girls accepted the situation, and when their parents died, degenerated into that saddest of all creatures, the "indigent gentlewoman." Now they are wiser; they prepare themselves for the possibilities of the future by taking up some line of work by which they may earn a living if ever need come. If they are wise they will not wait till the need has come, when there will be no money to train them for the profession they may desire to follow. This is the true reason why so many women are now seeking wage-earning work. Much of that work is ill-paid, and it may be safely said that in many instances it would pay better for the father to dismiss his servants and let his daughters do the housework. Needless to add, such a training is better for their future life, if that future holds for them what most parents desire for their daughters—a happy home of their own.

Why, then, is this course not more generally followed? For two reasons. In the first place, domestic work, though it may have a real money-value in the home, has not always a wage-earning value outside it. If a girl does not marry, to what is she to turn when her parents die? It is a fortunate thing that housekeeping is now beginning to be recognised as a profession. To be house-mistress in the boarding-house of a large school is felt to be a post which demands for its proper fulfilment the presence of an educated and refined, as well as capable woman, one who knows all about cookery, and laundry work, and general management, and also the something more that makes a home. That well-bred and well-educated women are going into such work is one of the most wholesome signs of the times. But such posts are as yet few. The ordinary home-bred girl may not be able to obtain a place like this, and what is she to do? The lady-help is beginning to be something more than merely a joke for the comic papers, but however willing a lady may be to do domestic work, she is not ready to be regarded by her employer as belonging to an inferior class. And if mistresses want to get rid of the eternal domestic problem, by employing women of superior quality, they must be prepared to treat them with as much consideration as they would a secretary. Moreover—and this is the second reason why ladies are unwilling to undertake domestic work—no lady likes dirty work. Therefore, if they are to be induced to undertake household duties there must be a much larger use of labour-saving inventions than as yet characterises the British household. If the heating of the house was done by a furnace in the cellar, and gas was used for cooking, the very unpleasant work of cleaning flues and polishing grates would be largely diminished. But will John Bull and Mrs. John Bull consent to give up those accustomed features of their home, the blazing fire and the kitchen range? If not, they must be content with the not too dainty service of non-fastidious Mary Jane. Will they give up the damp basement kitchen? Will they furnish the servant's room with neat furniture, instead of making it a lumber-room for all the cast-offs of other apartments? Will they, in short, treat their servants as ladies? If so, they will draw into the ranks of domestic work many who now go out to tasks for which they have less natural liking, and for which they are less fitted by nature. But so long as a convention exists that domestic work is unfitted for a lady, so long will those who are born and those who wish to be thought ladies keep out of it. They will naturally and rightly try to find work in positions which carry with them a certain amount of respect and consideration. The American writer, with whose opinion we started, does not think that women will rush from their typewriters, begging to be taken at any price into our kitchens. On the contrary, he believes that the result will be the putting of domestic service more on the footing of other businesses, that duties will be more clearly defined, competence in fulfilling them more firmly demanded; but as the idea that idleness and refinement go hand in hand vanishes more and more into the past, it will be accepted as a matter of course that there is nothing undignified in doing the work of the household, either in one's own home or in anyone else's.

